# The role of psychosocial factors in mediating the treatment response of epidural steroid injections for low back pain with or without lumbosacral radiculopathy: A scoping review

**DOI:** 10.1371/journal.pone.0316366

**Published:** 2025-01-15

**Authors:** Meredith Stensland, Donald McGeary, Caleigh Covell, Elizabeth Fitzgerald, Mahsa Mojallal, Selena Lugosi, Luke Lehman, Zachary McCormick, Paul Nabity

**Affiliations:** 1 Department of Psychiatry and Behavioral Sciences, University of Texas Health Science Center at San Antonio, San Antonio, TX, United States of America; 2 South Texas Veterans Health Care System, San Antonio, TX, United States of America; 3 Department of Psychology, University of Texas at San Antonio, San Antonio, TX, United States of America; 4 School of Medicine, University of Texas Health Science Center at San Antonio, San Antonio, TX, United States of America; 5 Department of Rehabilitation Medicine, University of Texas Health Science Center at San Antonio, San Antonio, TX, United States of America; 6 Department of Physical Medicine and Rehabilitation, University of Utah School of Medicine, Salt Lake City, UT, United States of America; National Institute of Child Health and Human Development (NICHD), NIH, UNITED STATES OF AMERICA

## Abstract

Epidural steroid injections (ESIs) are often used to treat low back pain (LBP) due to lumbosacral radiculopathy as well as LBP without a clear component of radiculopathy, in some cases. While it is increasingly recognized that psychosocial factors are associated with pain outcomes, few studies have assessed the contribution of these factors to common pain interventions like ESIs. This study aimed to summarize the scope and nature of how psychosocial factors are accounted for in research on ESIs for the treatment of LBP with or without lumbosacral radiculopathy and to identify gaps and recommendations for future research. A scoping review following the Preferred Reporting Items for Systematic Reviews and Meta-Analysis-Scoping Review Extension framework was conducted. Publications dated before September 2023 were searched in PubMed, CINAHL, Scopus, PsycINFO, and Google Scholar. Of the 544 records identified through database searching, a total of 51 studies cumulatively totaling 10,447 participants were included. Sample sizes ranged from 12 to 5,104 participants. Of the 51 included studies, only 10 (20%) analyzed and reported the relationship between at least one psychosocial variable and post-injection pain at any follow-up timepoint. The other 41 (80%) included no analyses examining ESI response as a function of psychosocial variables. Based on the studies that included analysis by psychosocial variables, poor psychosocial functioning appears to be associated with inferior treatments outcomes following ESI for back pain with or without lumbosacral radiculopathy. Relative to the vast body of literature on ESIs for LBP and lumbosacral radiculopathy, minimal attention has been directed to the influence of psychosocial factors on ESI treatment outcomes. Future research evaluating predictors of the effect of ESI on pain relief should include development of more comprehensive models containing modifiable psychosocial variables as predictors of ESI response.

## Introduction

Lumbosacral radiculopathy is one of the leading complaints evaluated by interventional pain physicians and spine surgeons alike [1]. Caused primarily by degenerative spondylosis with stenosis due to disc and/or bony sources [2], lumbar radiculopathy is characterized by pain in the lower back which radiates down to one or both legs. Compared to patients who have LBP *without* radiculopathy, those who also report radiculopathy have more severe pain intensity, increased somatosensory abnormalities, and more pronounced loss of small fiber function [3]. While prevalence estimates range from 3–5% of the general population, back pain with a radicular component accounts for a disproportionately high amount of the associated financial care costs; the average costs associated with an individual suffering from neuropathic back pain is 67% higher than that of a patient with nociceptive back pain only [4]. Lastly, lumbosacral radiculopathy is associated with high rates of disability [5] and decreased quality of life [6].

Epidural steroid injection (ESI) is the most common outpatient procedure performed for the treatment of spinal pain [7, 8], and it involves injecting one or more anti-inflammatory medications directly in the epidural space surrounding spinal nerves to address related neuropathic pain. Particularly for patients with radicular pain who have not responded to other moderate approaches, ESIs are increasingly being utilized. Between 2000 and 2011, ESI utilization grew 665% among Medicare beneficiaries alone [9]. Despite the dramatic increase in ESI utilization, important controversies remain with regard to efficacy within certainly sub-populations of patients with chronic LBP [10, 11] and cost-ineffectiveness [12]. Specifically for long-term pain improvement, past research is mixed with regard to the durability of the effect of ESIs, which must, at minimum, be repeated periodically to re-instate pain relief [13–20]. When pain relief is not achieved or sustained, some patients undergo repeat procedures or delay surgical interventions, depending on the etiology of lumbosacral radiculopathy, which results in additional utilization of health care resources [21, 22]. While ESIs are generally considered to have a favorable safety profile when performed according to clinical practice guidelines [23, 24], particularly when compared to opioid pain medications and surgery, they do carry potential risks including infection, spinal fluid leak, and other rare but non-trivial complications [25–27].

Concerns about the long-term efficacy and/or need for ongoing repeat ESIs in specific sub-populations coupled with the cost considerations and potential side effects of such procedures highlight the need for further understanding of the factors that influence patients’ response to ESIs. To date, research examining predictive factors for ESI response has focused heavily on electrodiagnostic [28–30] and imaging variables [31, 32], as well as route of injection [33], and agents injected [18, 19, 34, 35]. A large body of research consistently links psychosocial functioning with chronic pain intervention outcomes in general [36, 37], but there is scant research examining psychological factors as predictors of ESI outcomes despite promising studies published for other pain procedures. Studies on back surgery, for example, demonstrate that psychosocial variables play a critical role in how CLBP patients respond to surgical interventions. A recent review of 96 articles reported that among patients undergoing spine surgery, those with underlying psychological disease have longer hospital stays and higher rates of re-admission [38]. Other back surgery studies have shown that anxiety is associated with worse post-operative pain [39], depression increases the incidence of neurological complications and failed back surgery syndrome [40], and psychological distress predicts more severe post-operative disability [41]. Conversely, both pre-intervention [42, 43] and post-intervention [39] improvements in psychological distress are associated with better back surgery outcomes, so better understanding of psychosocial factors predicting ESI outcomes could lead to better treatment response with ESI.

Psychosocial factors may help explain differences in ESI response, however, these factors are infrequently studied and there is little consensus on their role in ESI. Thus, the purpose of this scoping review is to examine literature on the treatment response to ESI in individuals with LBP with or without lumbosacral radiculopathy, describe current knowledge about psychosocial factors and the role they play in ESI treatment response, note gaps in the literature, and discuss implications for future research.

## Methods

The present review followed the methodological procedures outlined by the Preferred Reporting Items for Systematic Reviews and Meta- Analysis Extension for Scoping Reviews [PRISMA-ScR] framework [44]. Scoping reviews are the preferred methodology when the objectives of the research involve determining the extent, range, and nature of the empirical evidence concerning a topic, along with identifying gaps in the literature [44]. Given our aims of examining the ESI literature for any evidence of the role that psychosocial factors play in treatment response, describing the expected weak state of this literature, and noting any significant gaps, scoping review is the ideal method. The literature search was performed electronically using relevant databases: PubMed, CINAHL, Scopus, PsycINFO, and Google Scholar. The search strategy, which was completed in September 2023, was developed iteratively by the research team in conjunction with an experienced health sciences librarian and encompassed both Medical Subject Headings terms and text words. The objective of this search was to capture studies reporting pain outcomes following ESI for the treatment of chronic LBP with or without lumbosacral radiculopathy, and which also included a focus on psychosocial factors.

Throughout the entire process of the scoping review, including screening, selection, and data extraction, six authors worked in dyads, such that three experienced pain researchers/faculty members were each paired with three PhD psychology interns, respectively. During the initial screening, each individual reviewed the title and abstracts of the articles independently. Next, each dyad met to compare and contrast screening decisions and rationales. In the event there was disagreement between the two dyad members, they worked collaboratively to examine and explain each person’s respective decision. This included reading the abstract together and discussing the basis for one’s opinion. If, after this discussion, the disagreement was not resolved, the issue was then brought to the whole group of six authors. This same process was followed for the full-text review phase. Any disagreements on the inclusion of studies were resolved through consensus as a group, wherein the authors met together, discussed rationales for their opinions, and adjudicated any disagreements. Had the authors continued to disagree, a qualified third-party individual would have been invited to consult.

Inclusion criteria for the review were as follows: collection of empirical data on ESI, the ESI is for the treatment of neuropathic pain related to the lower back (lumbosacral) region, both pre-injection and post-injection pain intensity scores are reported, at least one psychosocial measure reported, any age range for sample, any timeframe for publication date, published in English, and full text available. For this search, “psychosocial variable” meant any measure related to the following factors: anxiety, depression, affect, quality of life, sleep/insomnia, stress, mood, outcome expectancy, self-efficacy, and essentially any DSM-V condition. We chose these specific operational definitions of “psychosocial variables” because they can be easily assessed, are relevant to pain experience, and are modifiable (i.e., can be changed pre-ESI with some intervention). Exclusion criteria included the following: qualitative studies, review papers, animal or cadaver studies, studies that only examined ESI in combination with another intervention (e.g., medication, physical rehabilitation, educational intervention, etc.), studies that assessed an epidural injection without steroid, ESI for pain in the cervical or thoracic regions rather than the lower back, studies that did not have both pre and post-injection pain scores, and, most importantly, a lack of psychosocial measure(s) reported (See online Supplementary material file S1 File for full search strategy).

The first author developed a standardized data form for this review that facilitated the process of systematically extracting data from the included articles. The following data points were extracted: publication year, sample size, participants’ average age and standard deviation, proportion of male vs female, study design, route of epidural steroid injection, psychosocial variable(s) reported, pain measures, and relationship between psychosocial variables and pain. In cases where the study did not provide the mean age, the median or range was instead reported with estimated variances [45]. For studies that provided separate means and standard deviation for each treatment group (without a summary for the combined sample), the pooled average and standard deviation were reported. The extraction of data also followed the procedures previously outlined, such that it was first done independently, then discussed among the dyads, and then brought to the group as a whole on an as-needed basis. Study quality was assessed using the standardized Delphi list [46].

## Results

**Fig 1:**
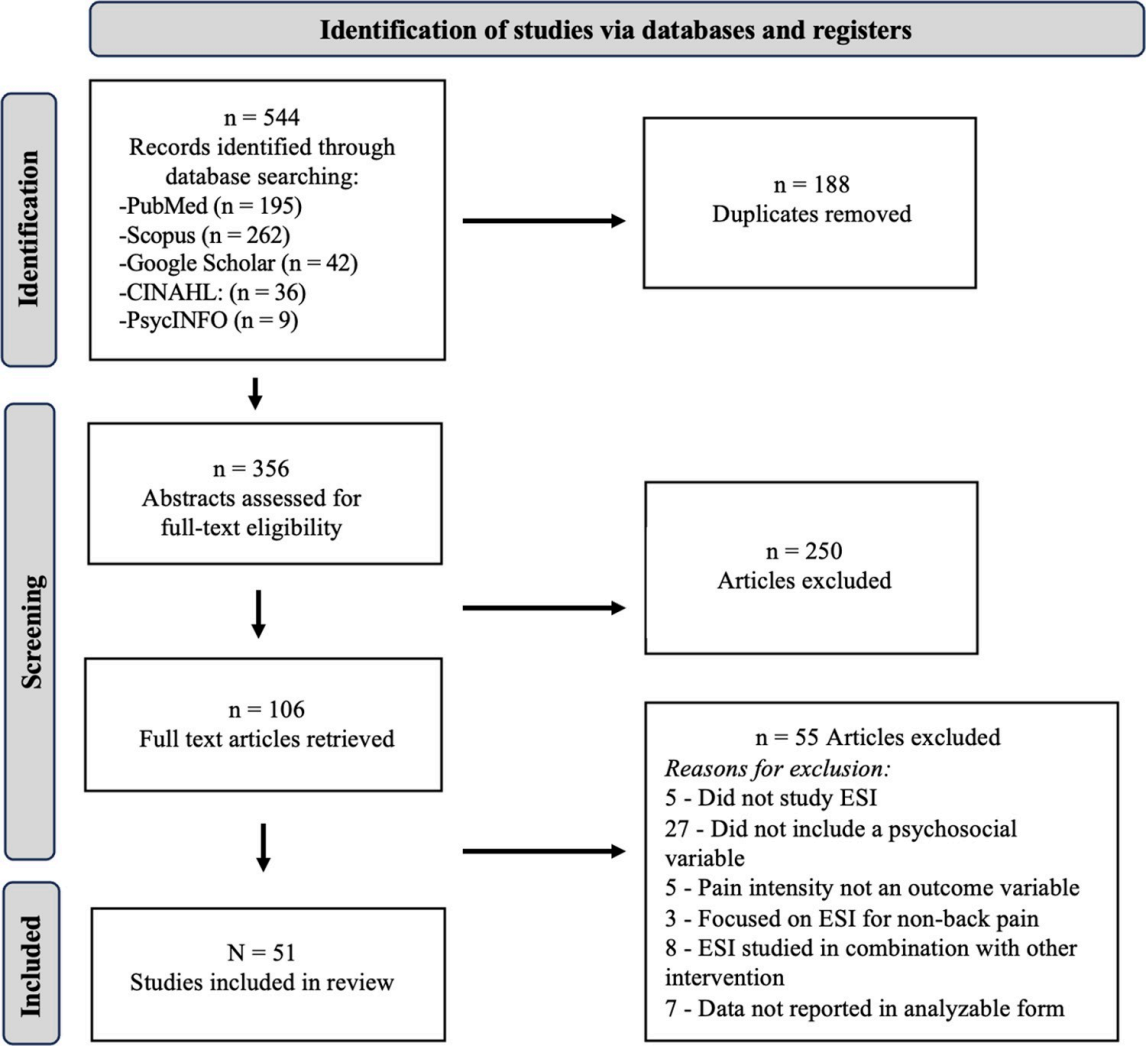
PRISMA flowchart of searching, screening, and selection methodology.

From the initial search, a total of 544 articles were identified prior to de-duplication: PubMed (195), Scopus (262), Google Scholar (42), CINAHL (36), PsycINFO (9). After duplicates were removed, a total of 356 articles were identified, and the title/abstracts were reviewed to assess suitability for full-text review. Following this step, a total of 106 full text articles were retrieved and assessed for relevance. Articles were excluded for a variety of reasons, including a focus on a spinal procedure other than ESI, a lack of psychosocial variables included, and the fact that pain was not an assessed outcome variable. Because the focus of this study was on chronic LBP and lower extremity radiculopathy, studies on ESIs for non-back pain (e.g., cervical or thoracic pain) were also excluded. Studies which examined ESI in combination with a different interventional procedure rather than ESI alone were also excluded, to avoid confounding findings. Ultimately, 51 studies met the inclusion criteria and were included in the present scoping review (see Fig 1).

### Study characteristics

The 51 included studies ranged in sample size from 12 to 5,104 totaling 10,447 participants. More than half of the studies (n = 27) reported an average (or median) sample age ≥ 50 years old. The following conditions were reported as the chief pain complaint: lumbar disc herniation, spinal stenosis, degenerative disc disease, nonspecific lower back pain, lumbosacral radiculopathy, sciatica, spondylolisthesis, and post-laminectomy syndrome. Studies implemented a variety of empirical research designs (see Table 1), including: Prospective observational (n = 27), Randomized controlled trial (n = 17), Retrospective observational (n = 4), and Other (n = 3). Routes of administration (type of injection) reported in the 51 studies included transforaminal (n = 16), interlaminar (n = 8) and caudal (n = 5), with n = 15 studies examining multiple injection routes and n = 7 that did not clearly report route of administration. The post-injection follow-up timepoints ranged from 15 minutes after the ESI was administered to 24 months post-injection.

**Table 1:** Description of study designs.

	Prospective observational	Retrospective observational	Randomized controlled trial	Other
Relationship between psychosocial variables and ESI outcomes not reported (n = 41)	19	4	16	2
Relationship between psychosocial variables and ESI outcomes reported (n = 10)	8	0	1	1
Total (n = 51)	27	4	17	3

### Psychosocial variables

A total of 19 different psychosocial variables were represented amongst the N = 51 included studies. Despite a large variety of variables represented, three constructs were most used: depression (n = 27), anxiety (n = 14), and quality of life (n = 10). Eight of the psychosocial variables identified in this scoping review were only examined in a single study (see Table 2).

**Table 2:** Psychosocial variables reported in the n = 51 studies.

Psychosocial Variable	Number of studies included in	Instruments(s) Used
Depression	27	HADS; QIDS; NRS; MZI; ZDI; PHQ; BDI; BDI (Korean Version); DASS; MCMI-II
Anxiety	14	HADS; PASS; MSPQ; GAD; DASS; STAI; PHQ; MCMI-II
Quality of Life	10	EQ-5D; WHOQOL
Treatment satisfaction	9	PSS; NASS; ZCQ; Yes/No; Likert
Health-related quality of life	8	SF-36; SF-12
Opioid use	8	MME; receipt of ≥1 opioid prescription in prior 12 months; number of opioid pills consumed daily; Yes/no; Internal rank order
Insomnia/sleep	7	ISI; PSQI; PROMIS; AIS; MOS; (“better, same, or worse”
Treatment expectations	4	NRS; Likert
Perceived overall functioning	3	SFMPQ; SF-36; NRS
Somatic sensory/perception	3	MCMI-II; SSAS; MSPQ
Fear avoidance	2	FABQ
Coping	1	PCQ
Psychological distress	1	DRAM
Negative affect	1	Composite of PROMIS and CES-D
Pain catastrophizing	1	PCS
Pain locus of control	1	PLOC
Mental health	1	NRS
Substance abuse	1	NR
Presence of any psychiatric condition	1	Clinical chart review

Abbreviations: AIS = Athens insomnia scale; BDI = Beck Depression Inventory; CES-D = Center for Epidemiological Studies Depression Scale; DASS = Depression Anxiety Stress Scales; DRAM = Distress and Risk Assessment Method; EQ-5D = EuroQol Quality of life index; FABQ = Fear Avoidance Beliefs Questionnaire; GAD = Generalized Anxiety Disorder scale; HADS = Hospital Anxiety and Depression Scale; ISI = Insomnia Severity Index; MCMI-II = Millon Clinical Multiaxial Inventory; MME = mean morphine equivalents; MOS = Medical Outcomes Study Sleep Scale; MSPQ = Modified Somatic Perception Questionnaire; MZI = Modified Zung index; NASS = North American Spine Society; NR = not reported; NRS = numeric rating scale; PASS = Pain Anxiety Symptom Scale; PCQ = Pain coping questionnaire; PCS = Pain Catastrophizing Scale; PHQ = Patient Health Questionnaire; PROMIS = Patient reported outcomes measurement information system; PSQI = Pittsburgh Sleep Quality Index; PSS = Patient Satisfaction Score; QIDS = Quick inventory of depressive symptomatology; SF-36 = Short Form-36; SFMPQ = Short Form McGill Pain Questionnaire; SSAS = Somatosensory amplification scale; STAI = State Train Anxiety Inventory; ZDI = Zung Depression Inventory; ZQC = Zurich Claudication Questionnaire.

While it was common for studies to utilize validated instruments to measure their psychosocial variables, unvalidated approaches were used in some cases, such as rating one’s own mental health on a basic, novel numeric rating scale or simply answering “Yes or No” rather completing a validated scale. Also, certain psychosocial variables were pulled from patients’ medical charts rather than being patient-reported directly. Further, some studies reported their data in a way that prevented them from being meaningfully analyzed. Examples of this issue ranged from stating that a psychosocial construct was administered but then never reporting any of the values for the measure, to reporting a composite score of many different factors, but not specifying the psychosocial subscale values.

### Relationship between psychosocial variables and post-injection pain severity

Of the 51 studies included in this review, a total of only 10 studies analyzed and reported on the relationship between at least one pre-injection psychosocial variable and post-injection pain at any follow-up timepoint. The longest duration of follow-up among these 10 studies was a 12-month post-injection timepoint. The extracted data from these 10 studies are summarized below in Table 3, and the summary table of the other 41 studies can be found online (see Supplementary S1 Table) [12, 16, 21, 47–84]. It should be noted that these other 41 studies reported on psychosocial variables but did not include any analyses explaining how the psychosocial variable is related to post-injection pain (the primary outcome of this review). Accordingly, this means that the large majority of the included studies collected patient-reported scores on psychosocial measures but did not include any of these variables as predictive factors for post-injection pain.

**Table 3:** Characteristics of studies examining the relationship between psychosocial variables and post-ESI pain.

Year and Author	Sample (size, sex, age)	Study Design	Psychosocial Variable(s) & Measure	Post-Injection Follow-up	Pain outcome measure	Type of injection	Relationship Between Psychosocial Variable(s) and Pain Outcome
Bahar-Ozdemir et al. (2020) [85]	N = 103 47%M/53%F 48.9 ± 13.4	Prospective observational	Depression (HADS) Anxiety (HADS) ∅ Somatic-sensory detection (SSAS) ∅	1 hour, 3 wk, 3 mo	NRS	TFESI	Greater depression symptoms, but not anxiety or SASS scores, were correlated with lower reduction of pain at both 3 weeks and 3 months post-injection.
Cohen et al. (2022) [86]	N = 346 53%M/47%F 52.0 ± 14.0	Prospective observational	Depression (QIDS) Insomnia (AIS) Expectation (6-pt Likert scale) ∅ Substance abuse (NR) Presence of any psychiatric condition (chart review) Opioid use (MME) ∅	1 mo, 3 mo	NRS	TFESI LESI	Greater depression, insomnia, substance abuse, and presence of any psychiatric condition all predictive of smaller pain reductions and treatment failure at follow-up.
Inman et al. (2004) [87]	N = 57 35%M/65%F 54.1 ± 1.5	Prospective observational	Anxiety (PASS) ∅ Pain coping (PCQ) Outcome Expectancy (NRS)	2 wk, 2 mo	NRS	NR	For female participants, emotion-focused avoidance coping correlated with more severe pain at both 2 weeks and 2 months, while problem-focused avoidance was correlated with less pain. Among males, problem focused avoidance predicted greater pain reductions at 2 months. More favorable outcome expectations were associated with lower pain intensity at two weeks, for females only. Anxiety was not correlated with pain.
Jindal et al. (2021) [88]	N = 96 16%M/84%F 49.3 ± NR	Prospective observational	Depression (MZI) ∅ Somatic perception (MSPQ) Psychological distress (DRAM) ∅	6 wk, 12 wk, 26 wk	VAS	CESI	Greater somatic perception predicted higher VAS at 6 weeks but not at 26 weeks. Change scores in depression and were not associated with changes in pain.
Joswig et al. (2016) [89]	N = 57 65%M/35%F 50 ± 10.7	Prospective observational	Health-related QOL (SF-12) Opioid use (yes/no) ∅	15 min, 30 min, 45 min, 1 hr, 2 hr, 4 hr, 1–14 days, 1 mo	VAS	TFESI	Baseline QOL higher in ESI responders vs. non-responders. No significant difference in rate of response between current opioid users and non-users.
Karp et al. (2014) [90]	N = 158 44%M/55%F 55.0 ± 13.6	Prospective observational	Negative affect (composite score of PROMIS and CES-D) ∅ Sleep (composite score of PROMIS and MOS)	1 mo, 3 mo	“Global report of back and/or leg pain”	LESI TFESI CESI	Sleep disturbance predictive of smaller pain improvements at both 1- and 3-months post-injection.
Kim et al. (2017) [91]	N = 161 61.3 ± 14.3 50%M/50%F	Prospective observational	Depression (ZDI)	3 mo, 1 yr	NRS	LESI TFESI CESI	Depressed group reported worse pain at baseline and less post-injection pain improvement compared to non-depressed participants.
Leblebicier et al. (2021) [92]	N = 36 53%M/47%F 44.0 ± 10.8	Prospective observational	Depression (BDI) ∅	1 mo, 3 mo	VAS	TFESI	Pre-injection BDI used to compare proportion of participants achieving ≥80% VAS improvement found no significant differences.
Mason et al. (2010) [93]	N = 133 35%M/65%F 55.9 ± 16.7	Prospective, non-randomized, wait control group	QOL (WHOQOL)	2 wk, 1 mo	VAS	NR	Psychosocial facets of WHOQOL (e.g., positive feelings, esteem, thinking/learning, negative feelings, anger, worry, frustration) demonstrated overall small effects, though were moderate for those reporting pain improvement. SD for the effects sizes were large, so finding is inconclusive.
Turner et al. (2015) [94]	N = 400 55%M/45%F 68.1 ± 10	RCT	Fear avoidance (FABQ) ∅ Pain Catastrophizing (PCS) ∅ Depression (PHQ-8) Anxiety (GAD-7) ∅ Treatment expectations (NRS 0–10) ∅ QOL (EQ-5D)	3 wk, 6 wk	NRS	LESI, TFESI	Greater depression predicts higher pain at 3 weeks; Higher QOL predicts lower pain at 6 weeks; all other psychosocial variables NS in ANCOVA analyses.

Note: ∅ = Not statistically significant predictor of/association with post-injection pain; AIS = Athens insomnia scale; ANOVA = analysis of variance; BDI = Beck Depression Inventory; CES-D = Center for Epidemiological Studies Depression Scale; CESI = caudal epidural steroid injection; DASS = Depression Anxiety Stress Scales; DRAM = Distress and Risk Assessment Method; EQ-5D = EuroQol Quality of life index; FABQ = Fear Avoidance Beliefs Questionnaire; GAD = Generalized Anxiety Disorder; HADS = Hospital Anxiety and Depression Scale; hr = hour; ISI = Insomnia Severity Index; LESI = interlaminar epidural steroid injection; MCID = minimal clinically important difference; MCMI-II = Millon Clinical Multiaxial Inventory; min = minute; MME = mean morphine equivalents; mo = month; MOS = Medical Outcomes Study Sleep Scale; MPQ = McGill Pain Questionnaire; MSPQ = Modified Somatic Perception Questionnaire; MZI = Modified Zung index; NASS = North American Spine Society; NR = not reported; NS = not statistically significant; PASS = Pain Anxiety Symptom Scale; PCQ = Pain coping questionnaire; PCS = Pain Catastrophizing Scale;; PHQ = Patient Health Questionnaire; PROMIS = Patient reported outcomes measurement information system; PSQI = Pittsburgh Sleep Quality Index; QIDS = Quick inventory of depressive symptomatology; RCT = randomized controlled trial; SF-36 = Short Form-36; SS = sample size; SSAS = Somatosensory amplification scale; STAI = State Train Anxiety Inventory; TFESI = transforaminal epidural steroid injection; VAS = visual analog scale; NRS = numeric rating scale; wk = week; yr = year; ZDI = Zung Depression Inventory; ZQC = Zurich Claudication Questionnaire.

Among the 10 main studies [85–94], there were a total of 28 instances in which a pre-injection psychosocial variable was analyzed in relation to post-injection pain, with the following results: n = 14 cases of worse psychosocial functioning being associated with worse pain; n = 0 cases of worse psychosocial functioning being associated with improved pain; and n = 14 cases in which psychosocial functioning and pain did not have a statistically significant relationship. Depression was directly analyzed in relation to pain in six studies; four of them found that more severe depression is associated with more severe pain or, inversely, smaller pain reductions [85, 86, 91, 94]. The other two studies found that the depression-pain relationship was not statistically significant [88, 92]. Anxiety was found to be not statistically significant in relation to pain for all three instances in which it was analyzed [85, 87, 94]. Both of the studies that examined sleep as a predictor of pain reported that sleep disturbance results in significantly smaller pain improvements at follow-up [86, 90]. Somatization variables were analyzed in relation to pain twice, with one study finding that greater somatic perception predicted higher pain scores at follow-up [88], and the other study reporting non-significance [85]. Both higher quality of life [93, 94] and health-related quality of life [89] are associated with lower pain scores. Of the three studies that examined pre-injection treatment expectations in relation to post-injection pain, one found that more favorable outcome expectations were associated with lower pain intensity at two weeks for female patients [87], while the other two studies reported non-significance between these two variables [86, 94]. Regarding prior opioid use, both studies that examined its use in relation to pain reported non-significance [86, 89].

Seven pre-injection psychosocial variables were analyzed in relation to post-injection pain on only one occasion. The following variables were found to be predictive of worse pain at follow-up: substance abuse [86], presence of any psychiatric condition [86], and maladaptive pain coping [87]. The following variables were found to be not significantly related to pain at follow-up: psychological distress [88], negative affect [90], pain catastrophizing [94], and fear avoidance [94].

It is important to note that while eight of these 10 main studies explicitly noted that the aim of the research was, at least in part, to examine pre-procedural patient-level individual or clinical characteristics (i.e., psychosocial variables) as predictors of ESI response, only two [86, 94] of these eight studies included an explicit explanation about having sufficient statistical power to address such an aim; these studies found that depression, insomnia, substance abuse, presence of any psychiatric condition, and low quality of life are all predictive of smaller pain reductions or treatment failure at follow-up after ESI. Regarding the measure of post-injection pain among these studies, the most common pain measures were Visual Analog and Numeric Pain Rating, both of which are validated, reputable measures for pain [95]. One study [90], however, used an unvalidated measure that was vaguely described as a “global report of back and/or leg pain.” It may be noted that the investigators examined and extracted a key procedural variable for this scoping review, type of injection (transforaminal, interlaminar, and caudal), however, there was no evidence from the reviewed studies showing an interaction between injection type and psychosocial variables (i.e., the role of depression symptom severity in outcomes of transforaminal versus interlaminar ESI) in their analyses of ESI outcomes.

### Study quality

The quality of the 10 studies that reported on the relationship of a pre-injection psychosocial variable with post-injection pain severity outcomes is presented in Table 4. Of these studies, only one was graded as a high quality randomized controlled trial [93]. There was one moderate quality wait list control group study [92], and the remaining eight trials were of lower quality (not highly controlled) prospective observational trials. These findings support the need for higher quality randomized controlled replication trials of ESI that include both pre-injection assessment of psychosocial variables and post-injection pain rating outcomes to develop reliable predictive models.

**Table 4:** Methodological quality of reviewed studies using Delphi list.

Publication	Design	1	2	3	4	5	6	7	8	9
Bahar-Ozdemir (2020) [85]	PO	—	—	—	Y	N	—	—	N	—
Cohen (2022) [86]	PO	—	—	—	Y	N	—	—	Y	—
Inman (2004) [87]	PO	—	—	—	Y	N	—	—	N	—
Jindal (2021) [88]	PO	—	—	—	Y	N	—	—	Y	—
Joswig (2016) [89]	PO	—	—	—	Y	N	—	—	Y	—
Karp (2014) [90]	PO	—	—	—	Y	N	—	—	N	—
Kim (2017) [91]	PO	—	—	—	Y	N	—	—	Y	—
Leblebicier (2021) [92]	PO	—	—	—	Y	N	—	—	Y	—
Mason (2010) [93]	PWCG	N	—	Y	Y	N	—	—	Y	—
Turner (2015) [94]	RCT	Y	Y	Y	Y	Y	Y	Y	Y	Y

Key. 1: Randomization performed; 2: Treatment allocation concealed; 3: Groups/subjects similar at baseline for important prognostic values; 4: Eligibility criteria specified; 5: Outcome assessor blinded; 6: Care provider blinded; 7: Patient blinded; 8: Point estimates and measures of variability presented for primary outcome measures; 9: Analysis included an intension-to-treat analysis. RCT = randomized controlled trial; PO = prospective observational; PWCG = Prospective wait control group Y = yes; N = no;—: not applicable.

## Discussion

This scoping review is the first study to comprehensively examine the empirical literature on the relationship between psychosocial variables and ESIs and for the treatment of chronic LBP with or without lumbosacral radiculopathy. A large body of research attempting to describe if patients achieve significant pain relief following ESI has been amassed over the last several decades, yet our review identified only 10 studies that explicitly analyzed the psychosocial-pain relationship in this context. Despite the field’s leading theoretical framework, the biopsychosocial model of pain [96], explicitly naming their importance, psychosocial variables appear to be only marginally represented in the ESI literature. This runs counter to a large body of past research consistently linking psychological factors with chronic LBP, including prevalent psychiatric co-morbidities [97] and maladaptive thinking patterns and behaviors tied to pain chronicity [98]. We argue that the gross underrepresentation of psychosocial factors in research on ESI, the most common outpatient procedure performed for back pain, is a critical oversight. As it stands, estimates of ESI efficacy are based on misspecified or incomplete models; the omission of important psychosocial variables likely introduces bias and skews the estimated effect of other predictors, thus failing to accurately capture the real relationship between the ESI procedure and patients’ treatment response. Our findings highlight a potential missed opportunity in understanding ESI treatment response (or non-response) and establishing screening or intervention methods to address psychosocial factors that could maximize ESI outcomes.

Amongst the 51 studies included in the present review, a total of 19 different psychosocial variables were identified. Depression, anxiety, and quality of life were the three most commonly used constructs. However, only roughly half of all the studies included in the review included a measure of depression (even fewer for anxiety) despite the availability of brief assessment options for these constructs and their broadly recognized importance in the field [99]. The 10 studies that analyzed and reported on the relationship between pre-injection psychosocial variables and post-injection pain at any follow-up timepoint indicated that poor psychosocial functioning was associated with *worse* ESI outcomes n = 14 cases and *improved* pain in n = zero cases; in n = 14 cases, psychosocial functioning and pain did not have a statistically significant relationship. While the reason for statistical insignificance varies by study, we point to instances of small sample size [87, 89, 92], use of invalidated or unknown measures (i.e., failure to report how something was measured [85], and poor methodological study quality. Most studies that examined a direct relationship specifically between depression and post-treatment pain outcomes showed significantly less pain improvement after ESI in individuals with greater depression symptoms. Only two studies did not find a relationship between depression and pain outcomes after ESI. However, one of these studies had a small sample size (N = 36), which may explain the lack of statistical significance observed. No study observed a relationship between poor psychosocial functioning and improved pain relief post-injection. Collectively, these findings suggest that the psychological state in which patients present to the clinic for their ESI may have important implications on their pain outcomes and considerations for treatment selection as well as concomitant psychosocial and medical interventions.

The consideration of psychosocial factors in interventions performed for chronic LBP is not a new concept. ESIs in their different approaches are the most commonly performed outpatient procedure for LBP with radiculopathy [100], but they are not the only intervention performed for this purpose. Spinal cord stimulation (SCS) is an additional, albeit more invasive and costly, procedural intervention often used in refractory cases of back pain, as well as other painful conditions. The relationship between psychosocial factors and SCS outcome has been extensively described and is compelling enough to lead many clinical services and third party payors to require psychological screening and evaluation as a prerequisite to be considered for this intervention [101–104]. Likewise, the spinal surgery literature has also made strides in accounting for psychosocial factors on surgical outcomes, demonstrating that greater presence of pre-surgical anxiety, depression, and psychological distress limit positive back surgery outcomes for CLBP patients specifically [39–41]. Pain outcomes related to psychosocial variables among SCS and surgical patients may provide a useful framework for which psychosocial variables to further study in the context of ESI response. Specifically, we recommend that the following psychosocial factors (and corresponding validated assessment tools) be considered in future work: depression (PHQ-9), anxiety (GAD-7), pan catastrophizing (PCS), and fear avoidance (FAB-Q). Not only are these variables consistently correlated with pain outcomes, but their inclusion across studies will facilitate future comparisons and synthesis of findings while likely strengthening predictive models by accounting for previously ignored mechanisms of response.

Given that the majority of the reviewed studies in this paper included no analysis of the relationship between psychosocial variables and post-injection pain, it is important to consider possible reasons for this underrepresentation of psychosocial factors. Historically, there has been an inherent bias in the medical research community whereby biological and physical factors are prioritized over psychological and social factors [105, 106]. It is only recently that biostatistical and theoretical frameworks have evolved to account for psychosocial functioning [107, 108]. It may also be noted that, by and large, research on ESIs is typically conducted by medical doctors who perform this procedure in their pain practice. It is possible that physicians’ greater experience with procedural relative to psychosocial variables, and possible motivation in some cases to demonstrate efficacy of the procedures they routinely perform, are driving the underrepresentation. Methodological challenges likely also impact the underrepresentation of psychosocial variables in ESI research. For example, psychosocial assessments may not be routinely performed in clinical pain management settings [109], so clinical datasets may not typically contain in-depth psychosocial measures. Thus, these assessments should be added into the normal flow of care prospectively, unlike procedural variables that occur as part of the routine ESI process (e.g., imaging, needle placement, etc.). The psychosocial assessments, depending on what is being administered, may be lengthy and consume a considerable amount of time; this can potentially introduce logistical challenges or cause assessment burden for the patients, the latter of which may increase risk of study attrition. However, recently evolutions in psychosocial assessment have led to much briefer options (cf. PHQ-4) [110]. In conclusion, we surmise that researcher biases, methodological challenges, and lack of behavioral investigators in this area are driving the underrepresentation of psychosocial factors in ESI research. It is our hope that future research will trend in the right direction and increasingly recognize the predictive value of including psychosocial assessments in ESI research, and, eventually, clinical care.

In line with the biopsychosocial model of pain, our review highlights the increasing need for research on individual differences that explain ESI patient outcomes, particularly in light of the documented variability and mixed findings regarding ESI effectiveness [13–16]. We highly encourage researchers to include assessments of psychosocial factors in future studies so that these critical variables can be appropriately included in statistical models of ESI treatment response. The inclusion of psychosocial variables in future ESI research supports precision medicine and could have a large impact on research and patient care [111]; equipped with an improved understanding of individuals’ likelihood of responding favorably to ESI, we can ensure those who are most likely to respond favorably receive the treatment, help connect those least likely to respond favorably to alternative therapies, and lower the rate of adverse events. Future studies may benefit from examining the role of potentially modifiable psychosocial outcomes, such as quality of life and mental health, on ESI outcomes. Additionally, these studies must be designed in such a manner and properly powered to ensures that the psychosocial variables are robustly tested as predictors. Understanding the impact of these psychosocial factors on pain outcomes is crucial for developing comprehensive treatment approaches that address both the physical and psychological aspects of back pain. Future research may also consider exploring the potential moderating and mediating roles of psychosocial factors in the relationship between ESI and pain relief. Additionally, investigating the mechanisms through which psychosocial variables affect ESI pain-related outcomes could provide valuable insights for clinical practice and the mechanisms of pain.

Because many of the discussed psychosocial variables are modifiable, it may be plausible that implementing a brief pre-injection intervention would increase a patient’s chance of responding well to ESI. This intervention could be rooted in cognitive-behavioral therapy, mindfulness, or relaxion techniques, all of which have shown promise in addressing the psychosocial factors covered in this scoping review [112–114]. With this approach, we can increase the volume of patients who benefit from the most commonly-performed outpatient back procedure in the United States, thereby optimizing patient care.

While we did not include self-reported disability in the present review, we note the importance of the field recognizing this important variable as a psychosocial predictor of ESI response in future research. The concept of disability itself is a subjective, socially-determined notion that can fluctuate over time [115]. Furthermore, self-reported disability is different than an objective physical test of functioning, as it simultaneously captures perceptions of one’s physical aspects, but also subjective appraisal of one’s emotional well-being [116]. Past research shows that self-reported disability is both highly-correlated with [117] and mediated by [118] psychosocial variables relevant to chronic pain. Understanding how patients perceive their own degree of pain-related disability may provide important psychosocial insights into the nature of their ESI response.

### Limitations

Although the PRISMA-ScR guidelines for scoping reviews were followed, [44] there are several limitations to note in the present study. Because the review was limited only to studies published in English, the comprehensiveness of the findings may be impacted. The authors presented a descriptive summary of the findings that does not explain the relationships, causes, or effects of findings. Furthermore, this scoping review included heterogeneous studies that utilized various interventions and assessment tools as well as heterogeneous samples, all of which may impact the interpretation of findings. The data extraction process is inherently subject to the possibility of selection bias, however, this was diminished via the use of a systematic approach and multiple authors reaching consensus on all decisions.

## Conclusion

ESI is the most common outpatient pain management procedure used for the treatment of LBP with or without lumbosacral radiculopathy, however, there are controversies regarding its efficacy when used within specific sub-populations. In order to better understand why results may vary, it is necessary to gain insight into what factors may affect patients’ response to ESI. Although studies on surgical procedures have supported the significance of psychosocial factors in treatment outcome, the existing literature on ESI has not sufficiently addressed the predictive role of pre-treatment psychosocial variables on treatment outcomes. The current findings highlight the need for further investigation of these variables in better understanding patients’ treatment response to ESI.

## Supporting information

S1 Checklist Preferred Reporting Items for Systematic reviews and Meta-Analyses extension for Scoping Reviews (PRISMA-ScR) checklist. (DOCX)

S1 Table Additional studies. Characteristics of studies that report on psychosocial variable(s) but did not include any analyses explaining how the psychosocial variable(s) are related to main outcome of post-injection pain (N = 41).
(DOCX)

S1 File Search syntax. Review strategy by database.
(PDF)

## References

[1] Berry JA, Elia C, Saini HS, Miulli DE. A review of lumbar radiculopathy, diagnosis, and treatment. *Cureus*. 2019;11(10): 1–7. doi: 10.7759/cureus.5934 31788391 PMC6858271

[2] Tarulli AW, Raynor EM. Lumbosacral radiculopathy. *Neurol Clin*. 2007;25(2):387–405. doi: 10.1016/j.ncl.2007.01.008 17445735

[3] Reimer M, Witthöft J, Greinacher J, Sachau J, Forstenpointner J, Hüllemann P, et al. Sensory profiles in patients with low back pain with and without radiculopathy. *Pain Med*. 2023;24(3):306–15. doi: 10.1093/pm/pnac129 36111863

[4] Schmidt CO, Schweikert B, Wenig CM, Schmidt U, Gockel U, Freynhagen R, et al. Modelling the prevalence and cost of back pain with neuropathic components in the general population. *Eur J Pain*. 2009;13(10):1030–5. doi: 10.1016/j.ejpain.2008.12.003 19201230

[5] Suri P, Carlson MJ, Rainville J. Nonoperative treatment for lumbosacral radiculopathy: What factors predict treatment failure? *Clin Orthop Relat Res*. 2015;473:1931–9. doi: 10.1007/s11999-014-3677-8 24832829 PMC4419012

[6] Golubovsky JL, Momin A, Thompson NR, Steinmetz MP. Understanding quality of life and treatment history of patients with Bertolotti syndrome compared with lumbosacral radiculopathy. *J Neurosurg Spine*. 2019:1–7. doi: 10.3171/2019.2.SPINE1953 31003219

[7] Schilling LS, Markman JD. Corticosteroids for pain of spinal origin. Epidural and intraarticular administration. *Rheum Dis Clin North Am*. 2016;42(1):137–55. doi: 10.1016/j.rdc.2015.08.003 26611556

[8] Manchikanti L, Pampati V, Parr A, Manchikanti MV, Sanapati MR, Kaye AD, et al. Cervical interlaminar epidural injections in the treatment of cervical disc herniation, post surgery syndrome, or discogenic pain: Cost utility analysis from randomized trials. *Pain Physician*. 2019;22(5):421–31. 31561644

[9] Manchikanti L, Pampati V, Falco FJ, Hirsch JA. Assessment of the growth of epidural injections in the medicare population from 2000 to 2011. *Pain Physician*. 2013;16(4):E349–64. 23877459

[10] Friedly JL, Comstock BA, Turner JA, Heagerty PJ, Deyo RA, Sullivan SD, et al. A randomized trial of epidural glucocorticoid injections for spinal stenosis. *N Engl J Med*. 2014;371(1):11–21. doi: 10.1056/NEJMoa1313265 24988555

[11] McCormick Z, Cushman D, Casey E, Garvan C, Kennedy DJ, Plastaras C. Factors associated with pain reduction after transforaminal epidural steroid injection for lumbosacral radicular pain. *Arch Phys Med Rehabil*. 2014;95(12):2350–6. doi: 10.1016/j.apmr.2014.07.404 25108099

[12] Pennington Z, Swanson MA, Lubelski D, Mehta V, Alvin MD, Fuhrman H, et al. Comparing the short-term cost-effectiveness of epidural steroid injections and medical management alone for discogenic lumbar radiculopathy. *Clin Neurol Neurosurg*. 2020;191:105675. doi: 10.1016/j.clineuro.2020.105675 31954364

[13] Yang S, Kim W, Kong HH, Do KH, Choi KH. Epidural steroid injection versus conservative treatment for patients with lumbosacral radicular pain: A meta-analysis of randomized controlled trials. *Medicine*. 2020;99(30). doi: 10.1097/MD.0000000000021283 32791709 PMC7386972

[14] Luijsterburg PA, Verhagen AP, Ostelo RW, Van Os TA, Peul WC, Koes BW. Effectiveness of conservative treatments for the lumbosacral radicular syndrome: A systematic review. *Eur Spine J*. 2007;16:881–99. doi: 10.1007/s00586-007-0367-1 17415595 PMC2219647

[15] Bresnahan BW, Rundell SD, Dagadakis MC, Sullivan SD, Jarvik JG, Nguyen H, et al. A systematic review to assess comparative effectiveness studies in epidural steroid injections for lumbar spinal stenosis and to estimate reimbursement amounts. *PM R*. 2013;5(8):705–14. doi: 10.1016/j.pmrj.2013.05.012 23953016

[16] Curatolo M, Rundell SD, Gold LS, Suri P, Friedly JL, Nedeljkovic SS, et al. Long‐term effectiveness of epidural steroid injections after new episodes of low back pain in older adults. *Eur J Pain*. 2022;26(7):1469–80. doi: 10.1002/ejp.1975 35604636 PMC9296573

[17] Ghahreman A, Ferch R, Bogduk N. The efficacy of transforaminal injection of steroids for the treatment of lumbar radicular pain. *Pain Med*. 2010;11(8):1149–68. doi: 10.1111/j.1526-4637.2010.00908.x 20704666

[18] McCormick ZL, Cushman D, Marshall B, Caldwell M, Patel J, Ghannad L, et al. Pain reduction and repeat injections after transforaminal epidural injection with particulate versus nonparticulate steroid for the treatment of chronic painful lumbosacral radiculopathy. *PM* *&R*. 2016;8(11):1039–45. 10.1016/j.pmrj.2016.03.011 27060648

[19] Kennedy DJ, Plastaras C, Casey E, Visco CJ, Rittenberg JD, Conrad B, et al. Comparative effectiveness of lumbar transforaminal epidural steroid injections with particulate versus nonparticulate corticosteroids for lumbar radicular pain due to intervertebral disc herniation: a prospective, randomized, double-blind trial. *Pain Med*. 2014;15(4):548–55. doi: 10.1111/pme.12325 24393129

[20] Smith CC, McCormick ZL, Mattie R, MacVicar J, Duszynski B, Stojanovic MP. The effectiveness of lumbar transforaminal injection of steroid for the treatment of radicular pain: A comprehensive review of the published data. *Pain Med*. 2020;21(3):472–87. doi: 10.1093/pm/pnz160 31343693

[21] Sivaganesan A, Chotai S, Parker SL, Asher AL, McGirt MJ, Devin CJ. Predictors of the efficacy of epidural steroid injections for structural lumbar degenerative pathology. *Spine J*. 2016;16(8):928–34. doi: 10.1016/j.spinee.2015.11.058 26689476

[22] Kennedy DJ, Zheng PZ, Smuck M, McCormick ZL, Huynh L, Schneider BJ. A minimum of 5-year follow-up after lumbar transforaminal epidural steroid injections in patients with lumbar radicular pain due to intervertebral disc herniation. *Spine J*. 2018;18(1):29–35. doi: 10.1016/j.spinee.2017.08.264 28962912

[23] Carr CM, Plastaras CT, Pingree MJ, Smuck M, Maus TP, Geske JR, et al. Immediate adverse events in interventional pain procedures: A multi-institutional study. *Pain Med* 2016;17(12):2155–61. doi: 10.1093/pm/pnw051 28025351

[24] El-Yahchouchi CA, Plastaras CT, Maus TP, Carr CM, McCormick ZL, Geske JR, et al. Adverse event rates associated with transforaminal and interlaminar epidural steroid injections: A multi-institutional study. *Pain Med*. 2016;17(2):239–47. doi: 10.1111/pme.12896 26593277

[25] D’Souza RS, Woods CD, Lee D, Kissoon N, Bendel M. Delayed cutaneous fluid leakage after fluoroscopic-guided epidural steroid injection. *Pain Med*. 2021;22(12):3092–5. doi: 10.1093/pm/pnab108 33755154

[26] Pountos I, Panteli M, Walters G, Bush D, Giannoudis PV. Safety of epidural corticosteroid injections. *Drugs R D*. 2016;16:19–34. doi: 10.1007/s40268-015-0119-3 26715572 PMC4767721

[27] Lee E, Lee JW, Kang HS. Interlaminar versus transforaminal epidural steroid injections: A review of efficacy and safety. *Skeletal Radiol*. 2023;52(10):1825–40. doi: 10.1007/s00256-022-04124-3 35859019

[28] Park DY, Kang S, Park JH. Factors predicting favorable short-term response to transforaminal epidural steroid injections for lumbosacral radiculopathy. *Medicina*. 2019;55(5):162. doi: 10.3390/medicina55050162 31109045 PMC6571939

[29] Meiling JB, Moman R, Pagan-Rosado R, Kinzelman-Vesely E, Hunt C, Hooten WM. Electromyography and therapeutic response to lumbosacral epidural steroid injections: A systematic review. *J Pain Res*. 2021:2851–8. doi: 10.2147/JPR.S327504 34539187 PMC8445100

[30] Annaswamy TM, Bierner SM, Chouteau W, Elliott AC. Needle electromyography predicts outcome after lumbar epidural steroid injection. *Muscle Nerve*. 2012;45(3):346–55. doi: 10.1002/mus.22320 22334168

[31] Cha SO, Jang CH, Hong JO, Park JS, Park JH. Use of magnetic resonance imaging to identify outcome predictors of caudal epidural steroid injections for lower lumbar radicular pain caused by a herniated disc. *Ann Rehabil Med*. 2014;38(6):791–8. doi: 10.5535/arm.2014.38.6.791 25566478 PMC4280375

[32] Ghahreman A, Bogduk N. Predictors of a favorable response to transforaminal injection of steroids in patients with lumbar radicular pain due to disc herniation. *Pain Med*. 2011;12(6):871–9. doi: 10.1111/j.1526-4637.2011.01116.x 21539702

[33] Lee JH, Kyoung-ho S, Park SJ, Lee GJ, Chang-Hyung L, Kim DH, et al. Comparison of clinical efficacy between transforaminal and interlaminar epidural injections in lumbosacral disc herniation: A systematic review and meta-analysis. *Pain Physician*. 2018;21(5):433. 30282389

[34] Tagowski M, Lewandowski Z, Hodler J, Spiegel T, Goerres GW. Pain reduction after lumbar epidural injections using particulate versus non-particulate steroids: Intensity of the baseline pain matters. *Eur Radiol*. 2019;29:3379–89. doi: 10.1007/s00330-019-06108-9 30887207

[35] McCormick Z, Kennedy DJ, Garvan C, Rivers E, Temme K, Margolis S, et al. Comparison of pain score reduction using triamcinolone vs. betamethasone in transforaminal epidural steroid injections for lumbosacral radicular pain. *Am J Phys Med Rehabil*. 2015;94(12):1058–64. doi: 10.1097/PHM.0000000000000296 25888660

[36] Hu Y, Yang Z, Li Y, Xu Y, Tian M, Jiang N, et al. Prevalence and associated factors of depressive symptoms among patients with chronic low back pain: A cross-sectional study. *Front Psychiatry*. 2022;12:820782. doi: 10.3389/fpsyt.2021.820782 35095623 PMC8793741

[37] Ranger TA, Cicuttini FM, Jensen TS, Manniche C, Heritier S, Urquhart DM. Catastrophization, fear of movement, anxiety, and depression are associated with persistent, severe low back pain and disability. *Spine J*. 2020;20(6):857–65. doi: 10.1016/j.spinee.2020.02.002 32045707

[38] Jackson KL, Rumley J, Griffith M, Agochukwu U, DeVine J. Correlating psychological comorbidities and outcomes after spine surgery. *Global Spine J*. 2020;10(7):929–39. doi: 10.1177/2192568219886595 32905726 PMC7485071

[39] Park C, Garcia AN, Cook C, Gottfried ON. Effect of change in preoperative depression/anxiety on patient outcomes following lumbar spine surgery. *Clin Neurol Neurosurg*. 2020;199:106312. doi: 10.1016/j.clineuro.2020.106312 33069091

[40] Schoell K, Wang C, D’Oro A, Heindel P, Lee L, Wang JC, et al. Depression increases the rates of neurological complications and failed back surgery syndrome in patients undergoing lumbar spine surgery. *Clin Spine Surg*. 2019;32(2):E78–E85. doi: 10.1097/BSD.0000000000000730 30346309

[41] Mancuso CA, Stal M, Duculan R, Girardi FP. Physical and psychological comorbidity independently associated with spine-related disability. *Spine*. 2014;39(23):1969–74. doi: 10.1097/BRS.0000000000000569 25365712

[42] Lee C-H, Liu J-T, Lin S-C, Hsu T-Y, Lin C-Y, Lin L-Y. Effects of educational intervention on state anxiety and pain in people undergoing spinal surgery: A randomized controlled trial. *Pain Manag Nurs*. 2018;19(2):163–71. doi: 10.1016/j.pmn.2017.08.004 29153299

[43] Davin SA, Savage J, Thompson NR, Schuster A, Darnall BD. Transforming standard of care for spine surgery: Integration of an online single-session behavioral pain management class for perioperative optimization. *Front Pain Res*. 2022;3:856252. doi: 10.3389/fpain.2022.856252 35599968 PMC9118343

[44] Tricco AC, Lillie E, Zarin W, O’Brien KK, Colquhoun H, Levac D, et al. PRISMA extension for scoping reviews (PRISMA-ScR): Checklist and explanation. *Ann Intern Med*. 2018;169(7):467–73. doi: 10.7326/M18-0850 30178033

[45] Hozo SP, Djulbegovic B, Hozo I. Estimating the mean and variance from the median, range, and the size of a sample. *BMC Med Res Methodol*. 2005;5(1):1–10. doi: 10.1186/1471-2288-5-13 15840177 PMC1097734

[46] Verhagen AP, De Vet HC, De Bie RA, Kessels AG, Boers M, Bouter LM, et al. The Delphi list: a criteria list for quality assessment of randomized clinical trials for conducting systematic reviews developed by Delphi consensus. *J Clin Epidemiol*. 1998;51(12):1235–41. doi: 10.1016/s0895-4356(98)00131-0 10086815

[47] Bahar Özdemir Y, Şencan S, Erçalık T, Kokar S, Gündüz OH. Do informative leaflets affect pre-procedural anxiety and immediate pain after transforaminal epidural steroid injections? A prospective randomized controlled study. *Agri*. 2021;33(1):1–6. doi: 10.14744/agri.2020.27048 34254651

[48] Batistaki C, Angelopoulou A, Smyrnioti ME, Kitsou MC, Kostopanagiotou G. Electromyographic findings after epidural steroid injections in patients with radicular low back pain: A prospective open-label study. *Anesth Pain Med*. 2017;7(6). doi: 10.5812/aapm.62556 29696128 PMC5903381

[49] Beyaz SG. Comparison of transforaminal and interlaminar epidural steroid injections for the treatment of chronic lumbar pain. *Braz J Anesthesiol*. 2017;67(1):21–7. doi: 10.1016/j.bjane.2015.06.003 28017166

[50] Brown LL. A double-blind, randomized, prospective study of epidural steroid injection vs. the mild® procedure in patients with symptomatic lumbar spinal stenosis. *Pain Pract*. 2012;12(5):333–41. doi: 10.1111/j.1533-2500.2011.00518.x 22272730

[51] Celenlıoglu AE, Sencan S, Gunduz OH. Does facet tropism negatively affect the response to transforaminal epidural steroid injections? A prospective clinical study. *Skeletal Radiol*. 2019;48(7):1051–8. doi: 10.1007/s00256-018-3129-8 30603772

[52] Dashfield AK, Taylor MB, Cleaver JS, Farrow D. Comparison of caudal steroid epidural with targeted steroid placement during spinal endoscopy for chronic sciatica: a prospective, randomized, double-blind trial. *Br J Anaesth*. 2005;94(4):514–9. doi: 10.1093/bja/aei084 15695544

[53] Erçalık T, Gencer Atalay K, Şanal Toprak C, Gündüz OH. Outcome measurement in patients with low back pain undergoing epidural steroid injection. *Turk J Phys Med Rehabil*. 2019;65(2):154–9. doi: 10.5606/tftrd.2019.2350 31453556 PMC6706822

[54] Hong J, Jung S. Clinical effectiveness and prognostic indicators of parasagittal interlaminar epidural injection. *Pain Physician*. 2016;19(6):E877–84. 27454278

[55] Joswig H, Neff A, Ruppert C, Hildebrandt G, Stienen MN. Repeat epidural steroid injections for radicular pain due to lumbar or cervical disc herniation: What happens after ‘salvage treatment’? *Bone Joint J*. 2018;100-b(10):1364–71. doi: 10.1302/0301-620X.100B10.BJJ-2018-0461.R1 30295524

[56] Kaur S, Gupta R, Singh S, Kumar R, Singh K. Impact of different approaches of epidural steroid injection on outcome of patients treated for low backache. *Anesth Essays Res*. 2017;11(3):697–701. doi: 10.4103/0259-1162.204205 28928574 PMC5594793

[57] Kircelli A, Cansever T, Yılmaz C. The influence of adjunctive caudal epidural steroid injection on the therapeutic effect of transforaminal epidural steroid injection. *Neurol India*. 2018;66(1):90–5. doi: 10.4103/0028-3886.222850 29322966

[58] Kvasnitskyi MV. Manifestations and treatment of lower back pain syndrome in wartime. Wiadomosci lekarskie (Warsaw, Poland: 1960). 2023;76(5 pt 2):1185–90. doi: 10.36740/WLek202305208 37364071

[59] Manchikanti L, Pampati V, Rivera JJ, Beyer C, Damron KS, Barnhill RC. Caudal epidural injections with sarapin or steroids in chronic low back pain. *Pain Physician*. 2001;4(4):322–35. 16902678

[60] Mendoza-Lattes S, Weiss A, Found E, Zimmerman B, Gao Y. Comparable effectiveness of caudal vs. trans-foraminal epidural steroid injections. *Iowa Orthop J*. 2009;29:91–6. 19742093 PMC2723700

[61] Ozsoy-Unubol T, Ercalik T, Gunduz OH. Comparison of epidural steroid injection efficiency with two different doses in radiculopathies associated with lumbar disc herniation. *World Neurosurg*. 2019. 10.1016/j.wneu.2018.12.056 30590213

[62] Park HS, Son YR, Choi KH. *Differences of therapeutic* responses to epidural steroid injection in elderly patients with radiculopathy. *Ann Geriat Med Res*. 2016;20(3):137–41. doi: 10.4235/agmr.2016.20.3.137

[63] Price C, Arden N, Coglan L, Rogers P. Cost-effectiveness and safety of epidural steroids in the management of sciatica. *Health Technol Assess*. 2005;9(33):iii–46. doi: 10.3310/hta9330 16095548

[64] Radoš I, Budrovac D, Ivan O, Černohorski H, Katarina Tot O, Drenjančević IH, et al. The influence of epidural steroids injections with transforaminal and interlaminal approaches on quality of sleeping, anxiety, and depression in patients with chronic lumbal radicular pain–prospective, randomized research. *Coll Antropol*. 2018;42(3):223–30.

[65] Ruiz-Lopez R, Tsai YC. A randomized double-blind controlled pilot study comparing leucocyte-rich platelet-rich plasma and corticosteroid in caudal epidural injection for complex chronic degenerative spinal pain. *Pain Pract*. 2020;20(6):639–46. doi: 10.1111/papr.12893 32255266

[66] Sarı S, Aydın ON, Güleser G, Kurt İ, Turan A. Effect of transforaminal anterior epidural steroid injection on neuropathic pain, quality of sleep and life. *Agri*. 2015;27(2):83–8. doi: 10.5505/agri.2015.91489 25944134

[67] Sari S, Aydın ON, Tasdemir B, Galimberti F, Turan A. Effect of statin use on pain relief by transforaminal epidural steroid injection. *Int J Med Res*. 2016;44(2):389–94. doi: 10.1177/0300060515597934 26912508 PMC5580055

[68] Sariyildiz MA, Batmaz İ, Yazmalar L, Güneş M, Turan Y. The effectiveness of transforaminal epidural steroid injections on radicular pain, functionality, psychological status and sleep quality in patients with lumbar disc herniation. *J Back Musculoskelet Rehabil*. 2017;30(2):265–70. doi: 10.3233/BMR-150438 27858682

[69] Sencan S, Celenlioglu AE, Yazici G, Gunduz OH. Transforaminal epidural steroid injection improves neuropathic pain in lumbar radiculopathy: A prospective, clinical study. *Neurol India*. 2021;69(4):910–5. doi: 10.4103/0028-3886.323894 34507411

[70] Sencan S, Edipoglu IS, Celenlioglu AE, Yolcu G, Gunduz OH. Comparison of treatment outcomes in lumbar central stenosis patients treated with epidural steroid injections: Interlaminar versus bilateral transforaminal approach. *Korean J Pain*. 2020;33(3):226–33. doi: 10.3344/kjp.2020.33.3.226 32606267 PMC7336349

[71] Serrao JM, Marks RL, Morley SJ, Goodchild CS. Intrathecal midazolam for the treatment of chronic mechanical low back pain: a controlled comparison with epidural steroid in a pilot study. *Pain*. 1992;48(1):5–12. doi: 10.1016/0304-3959(92)90125-U 1531383

[72] Shahgholi L, Yost KJ, Carter RE, Geske JR, Hagen CE, Amrami KK, et al. Correlation of the Patient Reported Outcomes Measurement Information System with legacy outcomes measures in assessment of response to lumbar transforaminal epidural steroid injections. *AJNR Am J Neuroradiol*. 2015;36(3):594–9. doi: 10.3174/ajnr.A4150 25614474 PMC8013067

[73] Spijker-Huiges A, Vermeulen K, Winters JC, van Wijhe M, van der Meer K. Epidural steroids for lumbosacral radicular syndrome compared to usual care: Quality of life and cost utility in general practice. *Arch Phys Med Rehabil*. 2015;96(3):381–7. doi: 10.1016/j.apmr.2014.10.017 25448243

[74] Tomkins-Lane CC, Conway J, Hepler C, Haig AJ. Changes in objectively measured physical activity (performance) after epidural steroid injection for lumbar spinal stenosis. *Arch Phys Med Rehabil*. 2012;93(11):2008–14. doi: 10.1016/j.apmr.2012.05.014 22659537

[75] Turan A, Sarwar S, Atim A, Deogaonkar A, Yousef HF, Katyal S, et al. Nitrous oxide for the treatment of chronic low back pain. *Anesth Analg*. 2015;121(5):1350–9. doi: 10.1213/ANE.0000000000000951 26484463

[76] Vaitkus A, Šipylaitė J. Qualitative sensory testing in outcome prediction of transforaminal epidural steroid injection for chronic painful unilateral lumbosacral radiculopathy: Prospective observational study. *Pain Pract*. 2021;21(6):618–29. doi: 10.1111/papr.12997 33502060

[77] Wilby MJ, Best A, Wood E, Burnside G, Bedson E, Short H, et al. Surgical microdiscectomy versus transforaminal epidural steroid injection in patients with sciatica secondary to herniated lumbar disc (NERVES): A phase 3, multicentre, open-label, randomised controlled trial and economic evaluation. *Lancet Rheumat*. 2021;3(5):e347–e56. doi: 10.1016/S2665-9913(21)00036-9 33969319 PMC8080892

[78] Yazici Sacaklidir G, Sencan S, Sacaklidir R, Gunduz OH. The effect of spinopelvic parameters on transforaminal epidural steroid injection treatment success in lumbar disc herniation. *Int J Clin Pract*. 2021;75(11):e14708. doi: 10.1111/ijcp.14708 34370361

[79] Godek P, Szczepanowska-Wolowiec B, Golicki D. GOLDIC therapy in degenerative lumbar spinal stenosis: Randomized, controlled trial. *Regen Med*. 2022;17(10):709–18. doi: 10.2217/rme-2022-0047 35899459

[80] Gupta R, Kaur H, Kaur S, Mahajan L, Kaur T. To compare the analgesic efficacy of two different doses of epidural ketamine in chronic low back-pain patients: A randomised double-blind study. *Indian J Anaesth*. 2020;64(9):768–73. doi: 10.4103/ija.IJA_541_20 33162571 PMC7641091

[81] Iversen T, Solberg TK, Romner B, Wilsgaard T, Twisk J, Anke A, et al. Effect of caudal epidural steroid or saline injection in chronic lumbar radiculopathy: Multicentre, blinded, randomised controlled trial. *BMJ*. 2011;343:d5278. doi: 10.1136/bmj.d5278 21914755 PMC3172149

[82] Adıgüzel E, Tecer D, Güzelküçük Ü, Taşkaynatan MA, Tan AK. The effectiveness of transforaminal epidural steroid injection in patients with radicular low back pain: Combination of pain provocation with effectiveness results. *Turk J Phys Med Rehabil*. 2017;63(2):117–23. doi: 10.5606/tftrd.2017.05882 31453439 PMC6648123

[83] Mhaskar VA, Pai S. Does the occupational activity level affect the quality of life of patients treated with epidural steroid injections for lumbar disc herniations? *Asian Spine J*. 2012;6(2):131–5. doi: 10.4184/asj.2012.6.2.131 22708017 PMC3372548

[84] Gharibo CG, Varlotta GP, Rhame EE, Liu EC, Bendo JA, Perloff MD. Interlaminar versus transforaminal epidural steroids for the treatment of subacute lumbar radicular pain: A randomized, blinded, prospective outcome study. *Pain Physician*. 2011;14(6):499–511. 22086091

[85] Bahar-Ozdemir Y, Sencan S, Ercalik T, Kokar S, Gunduz OH. The effect of pre-treatment depression, anxiety and somatization levels on transforaminal epidural steroid injection: A prospective observational study. *Pain Physician*. 2020;23(3):E273. 32517403

[86] Cohen SP, Doshi TL, Kurihara C, Reece D, Dolomisiewicz E, Phillips CR, et al. Multicenter study evaluating factors associated with treatment outcome for low back pain injections. *Reg Anesth Pain Med*. 2022;47(2):89–99. doi: 10.1136/rapm-2021-103247 34880117 PMC10327328

[87] Inman SL, Faut-Callahan M, Swanson BA, Fillingim RB. Sex differences in responses to epidural steroid injection for low back pain. *J Pain*. 2004;5(8):450–7. doi: 10.1016/j.jpain.2004.07.004 15501427

[88] Jindal R, Rudol G, Okafor B, Rambani R. Role of psychological distress screening in predicting the outcomes of epidural steroid injection in chronic low back pain. *J Clin Orthop Trauma*. 2021;19:26–33. doi: 10.1016/j.jcot.2021.04.027 34046297 PMC8141939

[89] Joswig H, Neff A, Ruppert C, Hildebrandt G, Stienen MN. The value of short-term pain relief in predicting the long-term outcome of lumbar transforaminal epidural steroid injections. *World Neurosurg*. 2016;107:764–71. doi: 10.1016/j.wneu.2017.08.055 28838872

[90] Karp JF, Yu L, Friedly J, Amtmann D, Pilkonis PA. Negative affect and sleep disturbance may be associated with response to epidural steroid injections for spine-related pain. *Arch Phys Med Rehabil*. 2014;95(2):309–15. doi: 10.1016/j.apmr.2013.09.007 24060493 PMC4008542

[91] Kim EJ, Chotai S, Stonko DP, Wick JB, Schneider BJ, McGirt MJ, et al. Patient-reported outcomes after lumbar epidural steroid injection for degenerative spine disease in depressed versus non-depressed patients. *Spine J*. 2017;17(4):511–7. doi: 10.1016/j.spinee.2016.10.017 27777051

[92] Leblebicier MA, Gündüz OH, Kaplan BM, Erçalık T. Role of paraspinal mapping before transforaminal epidural injections for lumbar radiculopathy. *Turk J Phys Med Rehabil*. 2021;67(2):196–202. doi: 10.5606/tftrd.2021.5042 34396070 PMC8343151

[93] Mason VL, Skevington SM, Osborn M. Assessing the properties of the WHOQOL-Pain: Quality of life of chronic low back pain patients during treatment. *Clin J Pain*. 2010;26(7):583–92. doi: 10.1097/AJP.0b013e3181e11369 20639736

[94] Turner JA, Comstock BA, Standaert CJ, Heagerty PJ, Jarvik JG, Deyo RA, et al. Can patient characteristics predict benefit from epidural corticosteroid injections for lumbar spinal stenosis symptoms? *Spine J*. 2015;15(11):2319–31. doi: 10.1016/j.spinee.2015.06.050 26096484

[95] Hawker GA, Mian S, Kendzerska T, French M. Measures of adult pain: Visual analog scale for pain (vas pain), numeric rating scale for pain (nrs pain), mcgill pain questionnaire (mpq), short‐form mcgill pain questionnaire (sf‐mpq), chronic pain grade scale (cpgs), short form‐36 bodily pain scale (sf‐36 bps), and measure of intermittent and constant osteoarthritis pain (icoap). *Arthritis Care Res*. 2011;63(S11):S240–S52. doi: 10.1002/acr.20543 22588748

[96] Gatchel RJ, Peng YB, Peters ML, Fuchs PN, Turk DC. The biopsychosocial approach to chronic pain: scientific advances and future directions. *Psychol Bull*. 2007;133(4):581. doi: 10.1037/0033-2909.133.4.581 17592957

[97] Reme SE, Tangen T, Moe T, Eriksen HR. Prevalence of psychiatric disorders in sick listed chronic low back pain patients. *Eur J Pain*. 2011;15(10):1075–80. doi: 10.1016/j.ejpain.2011.04.012 21592832

[98] Nieminen LK, Pyysalo LM, Kankaanpää MJ. Prognostic factors for pain chronicity in low back pain: A systematic review. *Pain Rep*. 2021;6(1):e919. doi: 10.1097/PR9.0000000000000919 33981936 PMC8108595

[99] Mullins PM, Yong RJ, Bhattacharyya N. Associations between chronic pain, anxiety, and depression among adults in the United States. *Pain Pract*. 2023;23(6):589–94. doi: 10.1111/papr.13220 36881021

[100] Johnson SM, Hutchins T, Peckham M, Anzai Y, Ryals E, Davidson HC, et al. Effects of implementing evidence-based appropriateness guidelines for epidural steroid injection in chronic low back pain: The EAGER (Esi Appropriateness GuidElines pRotocol) study. *BMJ Open Qual*. 2019;8(4):e000772. doi: 10.1136/bmjoq-2019-000772 31909212 PMC6937044

[101] Modak A, Jani R, Jani S, Mammis A. Psychiatric screening for spinal cord stimulation for complex regional pain syndrome: A literature review and practical recommendations for implementation. *Interdiscip Neurosurg*. 2023;31:101633. doi: 10.1016/j.inat.2022.101633

[102] Block AR, Ben-Porath YS, Marek RJ. Psychological risk factors for poor outcome of spine surgery and spinal cord stimulator implant: A review of the literature and their assessment with the MMPI-2-RF. *Clin Neuropsychol*. 2013;27(1):81–107. doi: 10.1080/13854046.2012.721007 22998432

[103] Beletsky A, Liu C, Alexander E, Hassanin SW, Vickery K, Loomba M, et al. The association of psychiatric comorbidities with short-term and long-term outcomes following spinal cord stimulator placement. *Neuromodulation*. 2023;26(5):1081–8. doi: 10.1016/j.neurom.2022.12.010 36720669

[104] Davis CE III, Kyle BN, Thorp J, Wu Q, Firnhaber J. Comparison of pain, functioning, coping, and psychological distress in patients with chronic low back pain evaluated for spinal cord stimulator implant or behavioral pain management. *Pain Med*. 2015;16(4):753–60. doi: 10.1111/pme.12526 25087848

[105] Ramtin S, Rajagopalan D, Ring D, Crijns T, Jayakumar P. Musculoskeletal surgeons have implicit bias towards the biomedical paradigm of human illness. *PloS one*. 2024;19(10):e0310119. doi: 10.1371/journal.pone.0310119 39446829 PMC11500957

[106] Hayes AM, Alpert E. Biomedical bias: The importance of countervailing information and multivariate models of risk and treatment of mental illness. *Clin Psychol*. 2017; 24(1), 74–77. doi: 10.1111/cpsp.12189

[107] Wade DT, Halligan PW. The biopsychosocial model of illness: A model whose time has come. *Clin Rehabil*. 2017; 31(8), 995–1004. doi: 10.1177/0269215517709890 28730890

[108] Woo SE, Hofmans J, Wille B, Tay L. Person-centered modeling: Techniques for studying associations between people rather than variables. *Annual Rev Organizational Psychol Organizational Behav*. 2024;11(1):453–80. doi: 10.1146/annurev-orgpsych-110721-045646

[109] Lee H-J, Choi EJ, Nahm FS, Yoon IY, Lee PB. Prevalence of unrecognized depression in patients with chronic pain without a history of psychiatric diseases. *Korean J Pain*. 2018;31(2):116–24. doi: 10.3344/kjp.2018.31.2.116 29686810 PMC5904346

[110] Kroenke K, Spitzer RL, Williams JB, Löwe B. An ultra-brief screening scale for anxiety and depression: the PHQ–4. *Psychosomatics*. 2009;50(6):613–21. doi: 10.1176/appi.psy.50.6.613 19996233

[111] Chadwick A, Frazier A, Khan TW, Young E. Understanding the psychological, physiological, and genetic factors affecting precision pain medicine: a narrative review. *J Pain Res*. 2021:3145–61. doi: 10.2147/JPR.S320863 34675643 PMC8517910

[112] Danilov A, Danilov A, Barulin A, Kurushina O, Latysheva N. Interdisciplinary approach to chronic pain management. Postgraduate Medicine. 2020; 132(3): 5–9. doi: 10.1080/00325481.2020.1757305 32298161

[113] Majeed MH, Ali AA, Sudak DM. Mindfulness-based interventions for chronic pain: Evidence and applications. *Asian J Psychiatr*. 2018;32:79–83. doi: 10.1016/j.ajp.2017.11.025 29220782

[114] Vambheim SM, Kyllo TM, Hegland S, Bystad M. Relaxation techniques as an intervention for chronic pain: A systematic review of randomized controlled trials. *Heliyon*. 2021;7(8). doi: 10.1016/j.heliyon.2021.e07837 34485731 PMC8405991

[115] Benítez‐Silva H, Buchinsky M, Man Chan H, Cheidvasser S, Rust J. How large is the bias in self‐reported disability? *J Appl Econ*. 2004;19(6):649–70. doi: 10.1002/jae.797

[116] Fiksenbaum LM, Greenglass ER, Marques SR, Eaton J. A psychosocial model of functional disability. *Ageing Int*. 2005;30:278–95. doi: 10.1007/s12126-005-1016-9

[117] Meyer K, Tschopp A, Sprott H, Mannion AF. Association between catastrophizing and self-rated pain and disability in patients with chronic low back pain. *J Rehabil Med*. 2009;41(8):620–5. doi: 10.2340/16501977-0395 19565155

[118] Varela AJ, Van Asselt KW. The relationship between psychosocial factors and reported disability: The role of pain self-efficacy. *BMC Musculoskelet Disord*. 2022;23:1–14. doi: 10.1186/s12891-021-04955-6 34980069 PMC8725494

