# Supplementary material for: The role of psychosocial factors in mediating the treatment response of epidural steroid injections for low back pain with or without lumbosacral radiculopathy: A scoping review
Source: PLoS One. 2025 Jan 15;20(1):e0316366. doi: 10.1371/journal.pone.0316366 (PMC11734955; doi:10.1371/journal.pone.0316366)
Supplement: S1 Table — Characteristics of studies that report on psychosocial variable(s) but did not include any analyses explaining how the psychosocial variable(s) are related to main outcome of post-injection pain (N = 41). (DOCX) [file pone.0316366.s002.docx]

The role of psychosocial factors in mediating the treatment response

of epidural steroid injections for low back pain with or without lumbosacral radiculopathy: A scoping review

Supplementary Material

**S1 Table**. **Additional studies.** Characteristics of studies that report on psychosocial variable(s) but did not include any analyses explaining how the psychosocial variable(s) are related to main outcome of post-injection pain (N = 41).

| **Year and Author** | **Sample**  **(size, sex, age)** | **Study Design** | **Psychosocial Variable(s)**  **& Measure** | **Post-Injection Follow-up** | **Pain outcome measure** | **Type of injection** |
| --- | --- | --- | --- | --- | --- | --- |
| Adıgüzel  (2017) [82] | N = 62  61%M/39%F  45 ± NR | Prospective observational | Patient functioning  (SF-36)  Patient satisfaction  (4-pt Likert) | 2 wk, 12 wk | VAS | TFESI |
| Bahar-Özdemir (2021) [47] | N=166  47%M/53%F  48.9 ± 13.6 | RCT | Depression, Anxiety (HADS) | 1 hr | NRS | TFESI |
| Batistaki (2017) [48] | N=39  26%M/74%F  65.9 ± 12.5 | Prospective  observational | Depression, anxiety, stress  (DASS)  Anxiety (STAI) | 1 yr | VAS | LESI |
| Beyaz  (2017) [49] | N=299  30%M/70%F  54.7 ± 11.69 | Prospective  observational | Satisfaction (NASS) | 1 mo ,3 mo, 6 mo, 9 mo, 1 yr | NRS | LESI  TFESI |
| Brown  (2012) [50] | N=38  55%M/45%F  76.2 ± 9.3 | RCT | Satisfaction (ZCQ) | 6 wk, 12 wk | VAS | LESI |
| Celenlıoglu (2019) [51] | N=100  41%M/59%F  44.7 ± 11.0 | Prospective observational | Depression (BDI) | 1 hr, 3 wk, 3 mo | NRS | TFESI |
| Curatolo  (2022) [16] | N=5104  33%M/67%F  73.8 ± 6.9 | Matched prospective observational | Depression, Anxiety (PHQ-4)  QOL (EQ-5D)  Patient expectation (NRS)^  Opioid use (receipt of  ≥1 opioid prescription in prior 12 months)^ | 3 mo, 6 mo, 1 yr, 2 yr | NRS | LESI  TFESI |
| Dashfield  (2005) [52] | N=60  45%M/55%F  46.3 ± 50.6 | RCT | Depression, Anxiety (HADS) | 6 wk, 3 mo, 6 mo | VAS | CESI |
| Erçalık  (2019) [53] | N=82  38%M/62%F  50.8 ± 14.2 | Prospective observational | Health-related QOL (SF-36) | 3 wk, 3 mo | NRS | TFESI  CESI |
| Gharibo  (2011) [84] | N=38  48%M/52%F  49.5 ± 14.7 | RCT | Depression (NRS)  Opioid use (number of pills consumed daily) | 10-16 days | NRS | LESI  TFESI |
| Godek  (2022) [79] | N = 90  28%M/72%F  70.1 ± 10.03 | RCT | QOL (EQ-5D) | 4 wk, 12 wk, 24 wk | NRS | LESI |
| Gupta  (2020) [80] | N = 60  52%M/48%F  48.37 ± 15.61 | RCT | Depression (NR)  Satisfaction (PSS) | 2 wk, 4 wk, 8 wk, 12 wk | VAS | LESI |
| Hong  (2016) [54] | N=55  42%M/58%F  61.4 ± 13.1 | Prospective observational | Depression (BDI Korean version) | 2 wk | NRS | LESI |
| Iversen  (2011) [81] | N = 116  59%M/41%F  41.9 ± 10.3 | RCT | QOL (EQ-5D)  Fear avoidance (FABQ)  Analgesic use  (yes/no) | 6 wk,12 wk, 1 yr | VAS | CESI |
| Joswig  (2018) [55] | N=12  67%M/33%F  54.2 ± 7.8 | Prospective observational | Health related QOL (SF-12)  Satisfaction  (5-pt Likert)  Opioid use  (yes/no) | 1 yr | VAS | TFESI |
| Kaur  (2017) [56] | N = 60  57%M/43%F  48.5 ± 14.8 | RCT | Depression (NR) | 1 wk, 1 mo, 3 mo | VAS | LESI  TFESI |
| Kircelli  (2018) [57] | N = 104  27%M/73%F  60.0 ± 15.6 | Retrospective observational | QOL (EQ-5D)  Satisfaction (NASS) | 1 mo, 6 mo, 1 yr | NRS | TFESI  CESI |
| Kvasnitskyi (2023) [58] | N = 120  %M vs %F NR  36-83 years | Retrospective observational | Anxiety (SHAT) | 3 mo | VAS | NR |
| Manchikanti (2001) [59] | N = 65  42%M/58%F  48.7 ± 15.8 | Prospective, non-randomized, with control group | Depression (BDI)  Mental health (0 to 10 self-rating)  Functioning (0 to 10 self-rating)  Narcotic intake (internal rank order measure)  Depression, Anxiety, Somatization, Symptom magnification  (MCMI-II) | 3 mo, 6 mo, 1 yr, 2 yr | NRS | CESI |
| Mendoza-Lattes  (2009) [60] | N = 93  %M vs %F NR  39.0 ± 13.1 | Retrospective observational | Health-related QOL (SF-36) | 6 mo, 1 yr, 2 yr | VAS | CESI  TFESI |
| Mhaskar  (2012) [83] | N = 90  M%/F% NR  20-65 years | Prospective observational | Health-related QOL (SF-36) | 2 wk, 1 mo, 6 mo | VAS | LESI |
| Ozsoy-Unubol  (2019) [61] | N = 67  30%M/70%F  49.7 ± 13.7 | Retrospective  observational | Health related QOL (SF-36) | 1 hr, 3 wk, 3 mo | NRS | TFESI |
| Park  (2016) [62] | N = 20  60%M/40%F  62.4 ± 13.0 | Prospective observational | Depression (BDI)  Insomnia (ISI) | 2 wk | VAS | LESI |
| Pennington (2020) [12] | N = 141  61%M/39%F  68.5 ± 10.5 | Prospective  observational | Depression (PHQ)  QOL (EQ-5D)  Opioid use  (NR) | 3 mo, 6 mo | VAS | TFESI  LESI |
| Price  (2005) [63] | N = 228  53%M/47%F  43.5 ± 12.0 | RCT | Depression, Anxiety (HADS)  Health related QOL (SF-36) | 3 wk, 6 wk, 3 mo, 6 mo, 1 yr | VAS | LESI |
| Radoš  (2018) [64] | N = 70  36%M/64%F  18-80 years | RCT | Depression, Anxiety (HADS)  Sleep (PSQI) | Immediately before the second ESI, before the third ESI, 2 wk after the third ESI, 12 wk, 24 wk later | VAS | TFESI  LESI |
| Ruiz‐Lopez  (2020) [65] | N = 50  42%M/58%F  64.5 ± 12.8 | RCT | Health related QOL (SF-36) | 1 mo, 3 mo, 6 mo | VAS | CESI |
| Sarı  (2015) [66] | N = 102  49%M/51%F  53.3 ± 11.3 | Prospective observational | Sleep (PSQI)  Health related QOL (SF-12) | 3 mo | VAS | TFESI |
| Sari  (2016) [67] | N = 293  45%M/55%F  58.4 ± 14.6 | Prospective observational | Health related QOL (SF-36) | 1 mo, 3 mo, 6 mo | VAS | TFESI |
| Sariyildiz (2017) [68] | N = 75  48%M/52%F  46.4 ± 12.5 | Prospective observational | Depression, Anxiety (HADS)  Sleep (PSQI) | 2 wk, 1 yr | VAS | TFESI |
| Sencan  (2021) [69] | N = 61  57%M/43%F  41.6 ± 11.1 | Prospective observational | Depression (BDI) | 1 hr, 3 wk, 3 mo | NRS | TFESI |
| Sencan  (2020) [70] | N = 72  27%M/73%F  62.5 ± NR | RCT | Depression (BDI) | 1 hr, 3 wk, 3 mo | NRS | TFESI  LESI |
| Serrao  (1992) [71] | N = 28  32%M/68%F  45.8 ± NR | RCT | Depression, Anxiety (HADS)  Pain locus of control (PLOC)  Sleep quality (better, same, worse)  Functioning (SFMPQ) | 1 hr, 24 hr, 2 wk, 2 mo | VAS | NR |
| Shahgholi (2015) [72] | N = 199  51%M/49%F  63.1 ± 14.7 | Prospective observational | QOL (EQ-5D) | 3 mo, 6 mo | NRS | TFESI |
| Sivaganesan (2016) [21] | N = 239  60.6 ± 14.2  44%M/56%F | Prospective observational | QOL (EQ-5D)  Health-related QOL (SF-12)  Anxiety (MSPQ)  Depression (ZDI)  Satisfaction (NASS) | 3 mo | NRS | LESI  TFESI  CESI |
| Spijker-Huiges (2015) [73] | N = 50  48%M/52%F  44.3 ± 9.5 | RCT | Health-related QOL (SF-36) | 4 wk, 13 wk, 26 wk, 1 yr | NRS | Segmental |
| Tomkins-Lane  (2012) [74] | N = 17  53%M/47%F  70.1± 6.7 | Prospective observational | Health-related QOL (SF-36) | 1 wk | VAS | NR |
| Turan  (2015) [75] | N = 78  51%M/49%F  71.5 ± 18 | RCT | Health-related QOL (SF-12)  Satisfaction  (yes/no) | 3 mo | VAS | NR |
| Vaitkus  (2021) [76] | N =36  28%M/72%F  58.7 ± 10.3 | Prospective observational | Depression and anxiety (HADS) | 1 wk, 4 wk | NRS | TFESI |
| Wilby  (2021) [77] | N = 163  47%M/53%F  42.4 ± 9.3 | RCT | Treatment satisfaction (Likert 1-5)  QOL (5D-5L) | 18 wk, 30 wk, 42 wk, 54 wk | NRS | TFESI |
| Yazici Sacaklidir (2021) [78] | N = 58  59%M/41%F  42.0 ± 9.5 | Prospective observational | Depression (BDI) | 1 hr, 3 wk, 3 mo | NRS | TFESI |

Note: ^ = baseline only; AIS = Athens insomnia scale; BDI = Beck Depression Inventory; BPI = Brief pain inventory; CESI = caudal epidural steroid injection; DASS = Depression Anxiety Stress Scales; DRAM = Distress and Risk Assessment Method; EQ-5D = EuroQol Quality of life index; FABQ = Fear Avoidance Beliefs Questionnaire; GAD = Generalized Anxiety Disorder; HADS = Hospital Anxiety and Depression Scale; hr = hour; ISI = Insomnia Severity Index; LESI = interlaminar epidural steroid injection; MCID = minimal clinically important difference; MCMI-II = Millon Clinical Multiaxial Inventory; min = minute; mo = month; MSPQ = Modified Somatic Perception Questionnaire; MZI = Modified Zung index; NASS = North American Spine Society; NR = not reported; NS = not statistically significant; PASS = Pain Anxiety Symptom Scale; PCQ = Pain coping questionnaire; PCS = Pain Catastrophizing Scale; PDQ = Pain Disability Questionnaire; PHQ = Patient Health Questionnaire; PLOC = Pain locus of control; PSQI = Pittsburgh Sleep Quality Index; PSS = Patient Satisfaction Score; QIDS = Quick inventory of depressive symptomatology; RCT = randomized controlled trial; SF-36 = Short Form-36; SFMPQ = Short Form McGill Pain Questionnaire; SHAT = Speilberger-Hanin Anxiety Test; SS = sample size; SSAS = Somatosensory amplification scale; STAI = State Train Anxiety Inventory; TFESI =transforaminal epidural steroid injection; VAS = visual analog scale; NRS = numeric rating scale; wk = week; yr = year; ZDI = Zung Depression Inventory; ZCQ = Zurich Claudication Questionnaire.
