# Supplementary material for: The role of psychosocial factors in mediating the treatment response of epidural steroid injections for low back pain with or without lumbosacral radiculopathy: A scoping review
Source: PLoS One. 2025 Jan 15;20(1):e0316366. doi: 10.1371/journal.pone.0316366 (PMC11734955; doi:10.1371/journal.pone.0316366)
Supplement: S1 File — Review strategy by database. (PDF) [file pone.0316366.s003.pdf]

S1 File. Search Syntax. Review strategy by database.
PsychINFO

#	Query	Results
S16	S6 AND S12	9
S15	S13 OR S14	170,196
S14	drug use disorder OR alcohol abuse OR substance abuse	135,554
S13	(DE "Substance Related and Addictive Disorders" OR DE "Addiction" OR DE "Nonsubstance Related Addictions" OR DE "Substance Use Disorder") OR(DE "Substance Use Disorder" OR DE "Alcohol Use Disorder" OR DE "Cannabis Use Disorder" OR DE "Drug Abuse" OR DE "Drug Dependency" OR DE "Inhalant Abuse" OR DE "Opioid Use Disorder" OR DE "Tobacco Use Disorder")	89,004
S12	S7 OR S8 OR S10 OR S11	1,657,375
S11	TI post-traumatic stress disorder ORAB post-traumatic stress disorder ORTI stress OR AB stress OR TI quality of life OR AB quality of life OR TI well-being OR AB well-being	416,713
S10	(DE "Substance Induced Psychotic Disorders" OR DE "Alcohol Induced Psychotic Disorders") OR (DE "Sleep Wake Disorders" OR DE "Hypersomnia" OR DE "Insomnia" ORDE "Narcolepsy" OR DE "Parasomnias" OR DE "Sleep Apnea" OR DE "Sleep Arousal Disorders" ORDE "Sleep Terrors" OR DE "Sleepwalking")	27,666
S9	S7 OR S8	1,465,569
S8	TI anxiety OR AB anxiety OR TI anxious OR AB anxious OR TI behavioral OR AB behavioral OR TI depression OR AB depression OR TI depress* OR AB depress* OR TI distress OR AB distress OR TI emotion OR AB emotion OR TI mental-health OR AB mental-health OR TI mood OR AB mood OR TI psychiatric OR AB psychiatric OR TI "prognostic factors" OR AB "prognostic factors"	1,274,510
S7	("psychological factor") OR psychosocial OR COPING OR" affective factors" OR "pain expectation"	320,806
S6	S3 AND S5	63
S5	S1 OR S2 OR S4	108
S4	(DE "Injections") AND (DE "Steroids")	91
S3	(lumbar OR back) OR ("Low back pain") OR ("Lower extremity radiculopathy") OR sacral OR spinal	99,589
S2	MA Injections, Epidural AND MA Steroids	18
S1	Epidural Steroid Injection OR "Interlaminar Epidural Steroid Injection" OR "Transforaminal Epidural Steroid Injection" OR "Caudal Epidural Steroid Injection"	41

PubMed

#	Query	Results
10	((#6 OR #7) AND (english[Language])) NOT (("animals"[MeSH Terms] NOT "humans"[MeSH Terms]))	195
9	(#6 OR #7) AND (english[Language])	203
8	#6 OR #7	214
7	#3 AND #5	19
6	#3 AND #4	201
5	("Substance-Related Disorders"[Mesh]) OR (drug use disorder OR alcohol use OR substance abuse)	758,759
4	(psychological factor) OR psychosocial OR COPING OR "affective factors" OR "pain expectation" OR anxiety[Title/Abstract] OR anxious[Title/Abstract] OR behavioral[Title/Abstract] OR depression[Title/Abstract] OR depress*[Title/Abstract] OR distress[Title/Abstract] OR emotion[Title/Abstract] OR mental-health[Title/Abstract] OR mood[Title/Abstract] OR psychiatric[Title/Abstract] OR prognostic factors[Title/Abstract] OR PTSD[Title/Abstract] OR post-traumatic stress disorder OR stress[Title/Abstract] OR quality of life[Title/Abstract] OR well-being[Title/Abstract] OR psychoses, substance induced[MeSH Terms] OR psychoses[Title/Abstract] OR Sleep Disorders, Intrinsic[Mesh] OR Sleep Wake Disorders[Mesh] OR sleep disorder[tiab] OR sleep disturbance[tiab] OR insomnia[tiab] OR hypersomnia[tiab]	3,976,174
3	#1 AND #2	2,150
2	(lumbar OR back) OR (Low back pain) OR (Lower extremity radiculopathy) OR sacral OR spinal	774,415
1	(Epidural Steroid Injection OR Interlaminar Epidural Steroid Injection OR Transforaminal Epidural Steroid Injection OR Caudal Epidural Steroid Injection) OR (("Injections, Epidural"[Mesh]) AND ("Steroids"[Mesh]))	2,690

Cinahl

#	Query	Results
S43	S42 NOT (((MH "Animals+") OR (MH"Animal Studies") OR (TI "animalmodel*")) NOT (MH "human"))	36
S42	S39 OR S40 AND ENGLISH	37
S41	S39 OR S40	38
S40	S15 AND S38	2
S39	S15 AND S35	36
S38	S36 OR S37	205,845
S37	drug use disorder OR alcohol use OR substance abuse	78,966
S36	(MH "Substance Use Disorders+") OR "substance related disorders"	187,300
S35	S16 OR S17 OR S18 OR S19 OR S20 OR S21 OR S22 OR S23 OR S24 OR S25 OR S26 OR S27 OR S28 OR S29 OR S30 OR S31 OR S32 OR S33 OR S34	1,421,418
S34	(TI sleep disorder OR AB sleep disorder) OR (TI insomnia OR AB insomnia) OR (TI hypersomnia OR AB hypersomnia)	16,404
S33	(MH "Sleep-Wake Transition Disorders+") OR "sleep wake disorders"	211
S32	(MH "Sleep Disorders, Intrinsic") OR "intrinsic sleep disorders"	165
S31	(TI psychoses OR AB psychoses) OR (TI psychosis OR AB psychosis)	15,903
S30	(MH "Psychoses, Substance-Induced+") OR "substance induced psychoses"	1,089
S29	(TI mood OR AB mood) OR (TI psychiatric OR AB psychiatric) OR (TI prognostic factors OR AB prognostic factors) OR (TI stress OR AB stress) OR (TI quality of life OR AB quality of life) OR (TI well-being OR AB well-being)	485,238
S28	TI "mental health" OR AB "mental health"	142,169
S27	TI emotion OR AB emotion	34,277
S26	TI distress OR AB distress	64,906
S25	TI depress* OR AB depress*	187,269
S24	TI depression OR AB depression	154,366
S23	TI behavioral OR AB behavioral	125,735
S22	TI anxious OR AB anxious	7,518
S21	TI anxiety OR AB anxiety	106,854
S20	(MH "Treatment Related Pain") OR "pain expectation"	2,303
S19	"affective factors"	189
S18	(MH "Coping+") OR "coping"	73,352
S17	(MH "Psychosocial Aspects of Illness+") OR "psychosocial"	788,014
S16	"psychological factor"	161
S15	S5 AND S14	334
S14	S6 OR S7 OR S8 OR S13	28,135
S13	S9 OR S12	135
S12	S10 AND S11	135
S11	(MH "Lower Extremity") OR "lower extremity"	28,905
S10	(MH "Radiculopathy") OR "radiculopathy"	3,875
S9	"lower extremity radiculopathy"	13
S8	(MH "Low Back Pain") OR "low back pain"	28,019
S7	sacral OR spinal	14
S6	lumbar OR back	23
S5	S1 OR S4	1,267
S4	S2 AND S3	705
S3	(MH "Steroids+")	8,610
S2	(MH "Injections, Epidural+")	2,107
S1	epidural steroid injection OR interlaminar epidural steroid injection OR transforaminal epidural steroid injection OR caudal epidural steroid injection	1,019

Scopus

((TITLE-ABS-KEY (("psychological factor") OR psychosocial OR coping OR "affective factors" OR "pain expectation" OR anxiety OR anxious OR behavioral OR depression OR depress* OR distress OR emotion OR mental-health OR mood OR psychiatric OR "prognostic factors" OR ptsd OR "post-traumatic stress disorder" OR stress OR "quality of life" OR well-being OR "substance induced psychoses" OR psychoses OR "Intrinsic Sleep Disorders" OR "Sleep Wake Disorders" OR "sleep disorder" OR "sleep disturbance" OR insomnia OR hypersomnia)) AND (((TITLE-ABS-KEY (("Epidural Steroid Injection" OR "Interlaminar Epidural Steroid Injection" OR "Transforaminal Epidural Steroid Injection" OR "Caudal Epidural Steroid Injection")) OR TITLE-ABS-KEY (("epidural injection*" AND steroid*)))) AND (TITLE-ABS-KEY ((lumbar OR back) OR ("Low back pain") OR ("Lower extremity radiculopathy") OR sacral OR spinal)))) OR ((TITLE-ABS-KEY (("Substance-Related Disorders") OR ("drug use disorder" OR "alcohol use" OR "substance abuse"))) AND (((TITLE-ABS-KEY (("Epidural Steroid Injection" OR "Interlaminar Epidural Steroid Injection" OR "Transforaminal Epidural Steroid Injection" OR "Caudal Epidural Steroid Injection")) OR TITLE-ABS-KEY (("epidural injection*" AND steroid*)))) AND (TITLE-ABS-KEY ((lumbar OR back) OR ("Low back pain") OR ("Lower extremity radiculopathy") OR sacral OR spinal)))) AND NOT ((INDEXTERMS (animals OR animal)) AND NOT (INDEXTERMS (humans OR human))) AND (LIMIT-TO (LANGUAGE , "English")) = 262 results.

Google Scholar

Epidural steroid injection AND (psychological factors | coping | pain expectation | distress | mental health| sleep | emotion) = 42 results
